# The Role of Transcription in the Activation of a *Drosophila* Amplification Origin

**DOI:** 10.1534/g3.114.014050

**Published:** 2014-10-14

**Authors:** Brian L. Hua, Sharon Li, Terry L. Orr-Weaver

**Affiliations:** * Whitehead Institute, Massachusetts Institute of Technology, Cambridge, Massachusetts 02142; † Department of Biology, Massachusetts Institute of Technology, Cambridge, Massachusetts 02142

**Keywords:** DNA replication, gene amplification, replication initiation, *yellow-g2*, promoter deletion

## Abstract

The mechanisms that underlie metazoan DNA replication initiation, especially the connection between transcription and replication origin activation, are not well understood. To probe the role of transcription in origin activation, we exploited a specific replication origin in *Drosophila melanogaster* follicle cells, *ori62*, which coincides with the *yellow-g2* transcription unit and exhibits transcription-dependent origin firing. Within a 10-kb genomic fragment that contains *ori62* and is sufficient for amplification, RNA-sequencing analysis revealed that all detected RNAs mapped solely to the *yellow-g2* gene. To determine whether transcription is required in *cis* for *ori62* firing, we generated a set of tagged *yellow-g2* transgenes in which we could prevent local transcription across *ori62* by deletions in the *yellow-g2* promoter. Surprisingly, inhibition of *yellow-g2* transcription by promoter deletions did not affect *ori62* firing. Our results reveal that transcription in *cis* is not required for *ori62* firing, raising the possibility that a *trans*-acting factor is required specifically for the activation of *ori62*. This finding illustrates that a diversity of mechanisms can be used in the regulation of metazoan DNA replication initiation.

DNA replication initiation occurs at specific genomic sites called origins of replication, and proper activation of these origins is essential for the precise duplication of the genome in dividing cells. In eukaryotes, replication initiation first requires the loading of the minichromosome maintenance (MCM)2-7 replicative helicase complex to origins of replication through the cooperative activities of the origin recognition complex (ORC) and the replication initiation factors Cdt1 and Cdc6 (Costa *et al.* 2013). In metazoans, origins of replication are not defined by DNA sequence (Cvetic and Walter 2005), and only a relatively small number of metazoan origins have been characterized in detail. The genomic positions and temporal programs of metazoan replication origins have been determined within various cell types by genome-wide sequencing analysis (Gilbert 2010), but the mechanisms that regulate recruitment of replication initiation factors to origins of replication and how origins of replication are activated, especially in the context of development, remain poorly understood.

The *Drosophila* follicle cell gene amplification system provides a powerful model for the study of individual metazoan origins *in vivo* (Claycomb and Orr-Weaver 2005). *Drosophila* follicle cells are somatic cells that surround the developing oocyte in the egg chamber and secrete the components of the egg shell (Spradling 1993). During gene amplification, specific origins of replication at six distinct regions within the follicle cell genome (called *Drosophila* amplicons in follicle cells, or *DAFC*s) are repeatedly activated during follicle cell differentiation while whole-genome replication is shut off (Spradling and Mahowald 1980; Claycomb *et al.* 2004; Kim *et al.* 2011). Origin firing is followed by bidirectional fork progression, resulting in a gradient of about 100-kb of amplified DNA (Claycomb and Orr-Weaver 2005). Importantly, this gene-amplification process requires the same replication factors as used during the typical S phase (Tower 2004; Claycomb and Orr-Weaver 2005). Follicle cell gene amplification takes place during a relatively short developmental time period (7.5 hr) between stages 10B and 13 of egg chamber development. Moreover, origin firing during gene amplification is tightly coordinated with egg chamber development, allowing high temporal resolution of individual origin firing events (Claycomb *et al.* 2002). As we have begun to characterize individual amplification origins to delineate the parameters of origin activation, it has become apparent that metazoan origins of replication use a diversity of mechanisms to regulate origin firing (Orr-Weaver *et al.* 1989; Xie and Orr-Weaver 2008; Kim *et al.* 2011; Kim and Orr-Weaver 2011). In this study, we focus on the possible link between transcription and DNA replication initiation.

Genome-wide origin mapping studies reveal that most origins are influenced by an open chromatin structure and closely coincide with the transcription start sites of actively transcribed genes, suggesting a correlative link between transcription and DNA replication initiation (Lucas *et al.* 2007; Cadoret *et al.* 2008; Sequeira-Mendes *et al.* 2009; Hansen *et al.* 2010; Karnani *et al.* 2010; MacAlpine *et al.* 2010; Mesner *et al.* 2011; Dellino *et al.* 2013; Mesner *et al.* 2013). However, how local transcription affects origin activation remains largely unknown. Limited studies suggest that transcription may play an important local role at origins of replication in several metazoan systems, including specification of a replication origin by a local promoter element (Danis *et al.* 2004) and delineation of a replication origin boundary by a transcription-arrest sequence (Mesner and Hamlin 2005). In budding yeast, the transcription machinery itself plays important roles in DNA replication initiation. Yeast RNA polymerase II has been shown to anchor ORC to rDNA replication origins (Mayan 2013) and to directly interact with the MCM2-7 helicase complex (Holland *et al.* 2002; Gauthier *et al.* 2002). Taken together, these studies raise the possibility that transcription may play an important and direct role in metazoan origin activation.

Previously, we found that one specific amplicon, *DAFC-62D*, exhibits transcription-dependent origin activation (Xie and Orr-Weaver 2008). The *DAFC-62D* origin, *ori62*, undergoes one round of origin firing during stage 10B and a second round of firing during stage 13 of egg chamber development. *ori62* maps completely within the transcriptional unit of the *yellow-g2* gene, and *yellow-g2* is transcribed in a strict and short developmental time window in stage 12, interspersing the two rounds of origin firing at *DAFC-62D*. The second round of origin firing is transcription-dependent, as addition of the RNA polymerase II inhibitor α-amanitin blocks stage 13 origin firing. Furthermore, transcription is required to localize the MCM2-7 helicase complex to *ori62* to allow the second round of origin firing (Xie and Orr-Weaver 2008). The requirement of transcription is specific for *DAFC-62D*, because all other amplicon origins activate normally in the presence of α-amanitin (Figure 1). These findings suggested a model that transcription is *cis*-activating at *DAFC-62D* and is required locally to promote the second round of origin firing.

**Figure 1:**
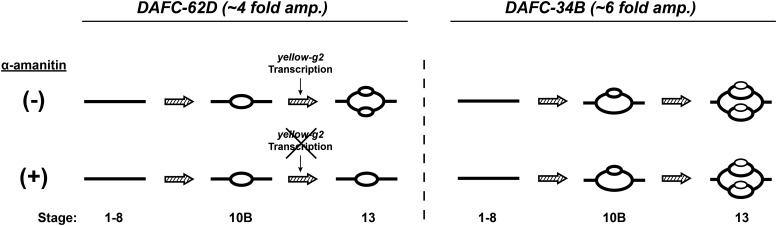
Left, Model of transcription-dependent origin activation of *DAFC-62D*. *DAFC-62D* exhibits two rounds of origin firing, the first at stage 10B and the second at stage 13 of egg chamber development, which are interspersed by transcription of the *yellow-g2* gene. In the presence of the RNA polymerase II inhibitor α-amanitin, stage 13 origin firing is specifically blocked (Xie and Orr-Weaver 2008). Right, Transcription-dependent origin firing is unique to *DAFC-62D*, as exemplified by normal origin firing of the comparable *DAFC-34B* in the presence of α-amanitin (Kim and Orr-Weaver 2011).

To test this model, we investigated the requirement of transcription specifically across *ori62* for the second round of *DAFC-62D* origin firing. First, we identified the RNAs present in follicle cells from a 10-kb region of *DAFC-62D* shown to be sufficient for amplification at ectopic insertion sites. We found that *yellow-g2* is the sole gene expressed in this minimal region. Therefore, we sought to delineate the role of local transcription in *ori62* activation by inhibiting transcription across *ori62* by *yellow-g2* promoter deletion. Our findings reveal that transcription in *cis* is not required for stage 13 *ori62* firing, suggesting the possibility of the requirement of a *trans*-acting factor for origin firing at *DAFC-62D*.

## Materials and Methods

### Generation of tagged genes

All constructs used in this study were derived from the PCRA10kb plasmid, which contains the 10-kb genomic locus corresponding to the central amplified region in *62D* previously described (Xie and Orr-Weaver 2008). To generate the tagged *yellow-g2* transgene, an *Apa*I/*Sac*II fragment containing the full *yellow-g2* coding sequence was subcloned into pBluescriptSK and a 21-bp tag sequence was inserted into the 3′ end of the *yellow-g2* coding sequence by site-directed mutagenesis to generate pBS-*Apa*I/*Sac*II-tag. A *Bgl*II/*Psh*AI fragment containing the tag was then liberated from this plasmid and used to replace the corresponding fragment in PCRA10kb to generate PCRA-10kb-tag. The *Not*I/*Avr*II fragment containing one Supressor of Hairy-wing binding site (*SHWBS*) and the 10-kb locus was then liberated and cloned into the *Not*I and *Nhe*I sites of the pCaSpeR4-*SHWBS P*-element transformation vector to generate pCaSpeR4-10kb-tag.

To generate the *yellow-g2* promoter deletions, a *Not*I/*Apa*I fragment containing a partial 5′ fragment of the *yellow-g2* coding sequence and 2.5-kb of upstream sequence was subcloned into pBluescriptSK to generate pBS-*Not*I/*Apa*I. Using site-directed mutagenesis, a 214-bp deletion was made to generate pBS-*Not*I/*Apa*I-Δ214, and a 1226-bp deletion was made to generate pBS-*Not*I/*Apa*I-Δ1226. The 214-bp deletion corresponds to the coordinates −120 to +94 relative to the transcription start site. The 1226-bp deletion corresponds to the coordinates −1132 to +94. The *Bbv*CI/*Apa*I fragment containing the promoter deletion was then liberated from each deletion construct to replace the corresponding fragment in PCRA-10kb-tag to generate PCRA-10kb-tag-214 and PCRA-10kb-tag-1226. The *Not*I/*Avr*II fragment containing one *SHWBS* and the 10-kb locus was then liberated from each plasmid and cloned into the *Not*I and *Nhe*I sites of the pCaSpeR4-*SHWBS P*-element transformation vector to generate pCaSpeR4-Δ214 and pCaSpeR4-Δ1226.

### Strains and transgenic lines

*P*-element transposon constructs were sent to BestGene Inc. (Chino Hills, CA) for individual injections into *w^1118^* embryos to establish at least two independent transformation lines per construct. To examine the effects of the Suppressor of Hairy-wing (Su(Hw)) chromatin insulator, transposons on the *2^nd^* chromosome were crossed into the *su(Hw)^2^Sb^sbd-2^/TM6*, *su(Hw)^5^* background.

### RNA isolation and RNA-seq

The RNA-seq analysis of total RNA extracted from purified 16C follicle cells has been described previously (Kim *et al.* 2011). In summary, RNA-seq libraries were generated using the mRNA-seq Sample Preparation kit from Illumina with the exception that RNAs were not poly(A)-selected. The RNA-seq library was size-selected for enrichment in the 200-nt range according to Illumina recommendation. This library was then subjected to Duplex-Specific Thermostable Nuclease treatment to remove highly abundant RNAs such as rRNAs and tRNAs.

### Transcription analysis

Total RNA was isolated from 30−50 stage 12 egg chambers using TRIzol reagent (Invitrogen). One microgram of total RNA was reverse transcribed using AMV reverse transcriptase (Promega) to generate single-stranded cDNA. Transgenic *yellow-g2* transcript levels were determined by quantitative polymerase chain reaction (qPCR) using total cDNA samples and a primer set specific for the tag within the transgenic *yellow-g2* transcript. In addition, a primer set that did not discriminate between the endogenous and transgenic *yellow-g2* transcripts was used to measure the total amount of *yellow-g2* transcript. Primer sequences are available on request. Each transcription analysis experiment was performed in biological duplicates.

### Amplification assay

Genomic DNA was isolated from staged egg chambers and quantified using relative qPCR as described (Xie and Orr-Weaver 2008). A primer set specific for the transposon boundary was used to assess transposon amplification, and a primer set specific for the corresponding endogenous site was used to assess endogenous *62D* amplification. Each DNA sample was internally normalized to the copy number of a nonamplified control locus at *93F*. Primer sequences are available on request. Fold amplification at a given developmental stage was determined relative to preamplification stage 1−8 egg chamber DNA. Each amplification assay was performed in biological triplicates.

## Results and Discussion

### *yellow-g2* is the sole transcription unit in the 10-kb *DAFC-62D* amplicon

**Figure 2:**
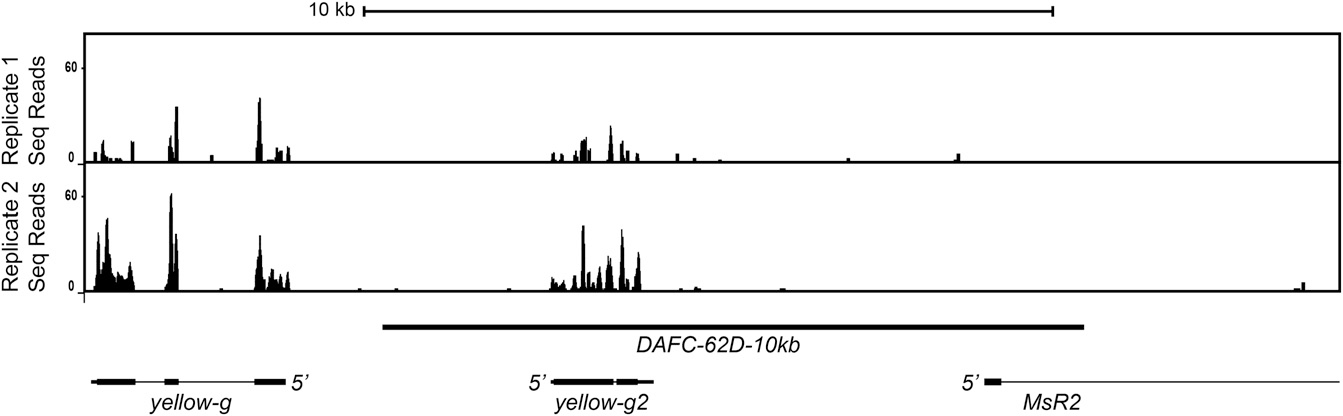
Analysis of transcripts within the 10-kb amplification-sufficient fragment of *DAFC-62D* (black bar) from two biological replicates. Total, non-poly(A)-selected RNA was isolated from 16C follicle cell nuclei, which are enriched for amplification stages. RNAs were sequenced and mapped to the *Drosophila* dm3 genome. The *chr3L*:2,270,000-2,288,000 region is shown, and the 5′ ends of each gene are depicted.

To examine the role of transcription in *ori62* firing, we first identified the RNAs within the 10-kb amplification-sufficient *DAFC-62D* region previously characterized (Xie and Orr-Weaver 2008). We isolated, sequenced, and mapped total, non-poly(A)-selected RNAs from 16C follicle cell nuclei, which are enriched for amplifying nuclei (Kim *et al.* 2011). Within this 10-kb region, nearly all RNAs mapped within the *yellow-g2* gene. The reads that mapped outside of the *yellow-g2* gene showed poor overlap in the two biological replicate experiments (Figure 2). We conclude that *yellow-g2* is the sole gene expressed in this region. Because the RNA-seq libraries we analyzed were size-selected for templates in the 200-nt range, we cannot exclude the possibility that small RNAs such as microRNAs exist in this 10-kb region.

### A tagged *yellow-g2* transgene

**Figure 3:**
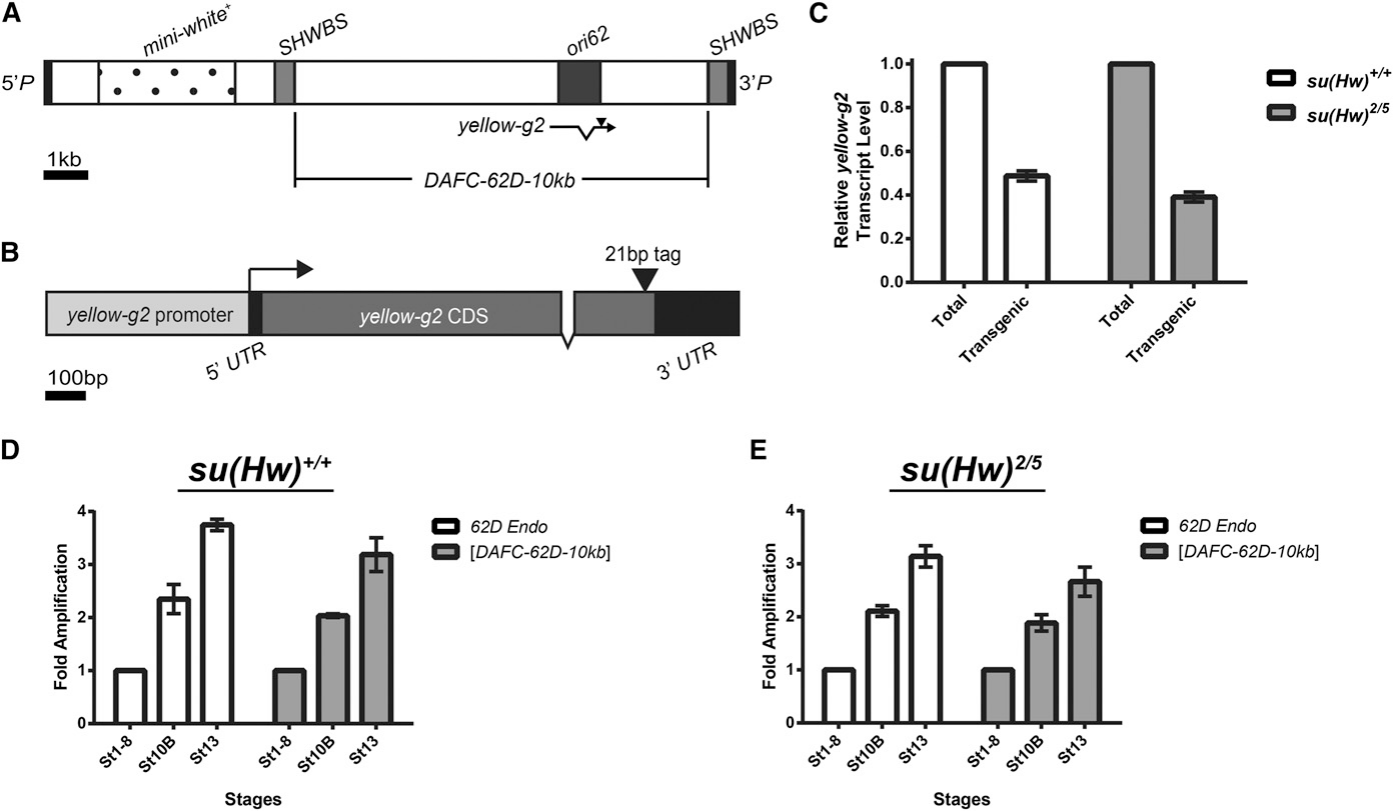
Characterization of the full-length tagged *yellow-g2* transgene. (A) Structure of the 16.2-kb [*DAFC-62D-10kb*] *P*-element transposon construct. (B) Diagram of the full-length, 1.5-kb *yellow-g2* transgene. A 21-bp tag was inserted in the 3′ end of the *yellow-g2* coding sequence. Extent of the full *yellow-g2* promoter is unknown. (C) Levels of transgenic *yellow-g2* transcripts relative to total *yellow-g2* transcripts isolated from stage 12 egg chambers in either wild-type or *su(Hw)* mutant backgrounds. Fold amplification of the endogenous *62D* locus (*62D Endo*) and the [*DAFC-62D-10kb*] transposon was measured in the wild-type (D) and the *su(Hw)* mutant (E) backgrounds. Error bars represent standard deviation of the mean of the biological triplicates.

To investigate the role of *yellow-g2* transcription in *ori62* activation, we used *P*-element−mediated transformation to generate 10-kb *DAFC-62D* transposon lines in which we could modulate *yellow-g2* transcription. The transposons were flanked by Suppressor of Hairy-wing (Su(Hw)) insulator binding sites (*SHWBS*) to protect from inhibitory position effects (Figure 3A) (Lu and Tower 1997).

To distinguish between the transgenic and endogenous *yellow-g2* gene, we inserted a short 21-bp tag at the 3′ end of the coding sequence of the *yellow-g2* transgene. First, we assessed whether the insertion of this tag affected *yellow-g2* transcription and transposon amplification. To this end, we generated a 10-kb *DAFC-62D* transposon that contained the full *yellow-g2* locus and the inserted tag and produced transgenic lines (Figure 3B). We then assessed transcription of the *yellow-g2* transgene relative to total *yellow-g2* transcription by isolating RNA from stage 12 egg chambers and performing reverse transcription followed by qPCR. Insertion of the 21-bp tag did not inhibit transcription of the *yellow-g2* transgene, as transgenic *yellow-g2* transcript was detectable and comprised about half the total *yellow-g2* transcript pool (Figure 3C). Next, genomic DNA was isolated from stage 1−8, 10B, and 13 egg chambers and genomic copy number of the transgene was measured by qPCR. Importantly, the tagged transgene amplified to normal levels and at the same developmental times as the endogenous *DAFC-62D*, as evidenced by twofold amplification in stage 10B egg chambers and 3- to 4-fold amplification in stage 13 egg chambers (Figure 3D). To confirm that the presence of the Su(Hw) insulator did not affect transgene transcription and amplification, we measured transcription and amplification of the *yellow-g2* transgene in wild-type and *su(Hw)* mutant backgrounds. We found that the *yellow-g2* transgene was transcribed and amplified to comparable levels in both the wild-type and *su(Hw)* mutant backgrounds (Figures 3, C and E, respectively). Taken together, insertion of the 21-bp tag into the *yellow-g2* transgene did not affect transcription or amplification of transgene.

### Transcription in *cis* is not required for *ori62* firing

**Figure 4:**
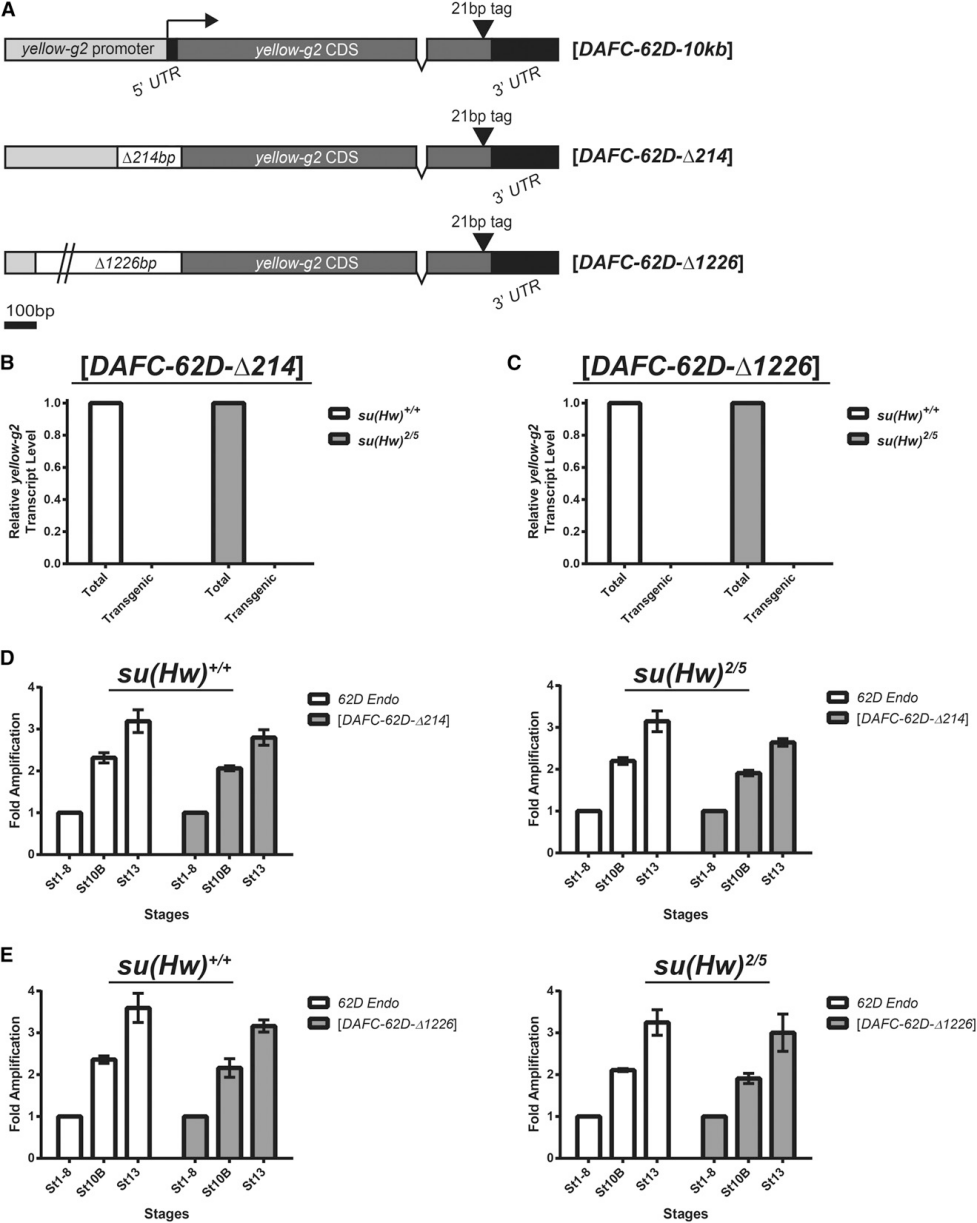
Characterization of the *yellow-g2* promoter deletion transgenes. (A) Diagrams of the full-length, 214-bp promoter deletion, and 1226-bp promoter deletion *yellow-g2* transgenes. The region deleted is shown in white. The 1226-bp deletion is not to scale. The extent of the full *yellow-g2* promoter is not known. Levels of transgenic *yellow-g2* transcripts relative to total *yellow-g2* transcripts isolated from stage 12 egg chambers were determined for the [*DAFC-62D-Δ214*] (B) and [*DAFC-62D-Δ1226*] (C) transgenic lines in either wild-type or *su(Hw)* mutant backgrounds. (D) Fold amplification of the endogenous *62D* locus (*62D Endo*) and the [*DAFC-62D-Δ214*] transposon in the wild-type and the *su(Hw)* mutant backgrounds. (E) Fold amplification of the endogenous *62D* locus (*62D Endo*) and the [*DAFC-62D-Δ1226*] transposon in the wild-type and the *su(Hw)* mutant backgrounds. Error bars represent standard deviation of the mean of the biological triplicates.

To test whether transcription is required in *cis* for *ori62* firing, we made two deletions in the promoter of the *yellow-g2* transgene to abrogate transcription across *ori62*. We generated transposon lines harboring the 10-kb *DAFC-62D* region with either a 214-bp or 1226-bp deletion of the *yellow-g2* promoter region (Figure 4A). Reverse-transcription qPCR analysis of RNA isolated from stage 12 egg chambers revealed that transcription of the *yellow-g2* transgene was inhibited completely by both promoter deletions, as transgenic *yellow-g2* transcript was not detected in either the 214-bp or the 1226-bp promoter deletion lines (Figure 4, B and C, respectively). Next, we isolated stage 1−8, 10B, and 13 egg chamber genomic DNA from each promoter deletion line to assess genomic copy number of the *yellow-g2* transgene. Surprisingly, we found that stage 13 origin firing still occurred despite inhibition of *yellow-g2* transcription across *ori62*, exhibiting 3- to 4-fold amplification in both the 214bp promoter deletion line (Figure 4D) and the 1226-bp promoter deletion line (Figure 4E). We observed comparable results in the wild-type and *su(Hw)* mutant backgrounds, confirming that our findings were not confounded by the presence of the Su(Hw) insulator. If transcription were required in *cis* for origin firing at stage 13, then inhibition of transcription of *yellow-g2* should have inhibited stage 13 origin firing. Thus, from these results we conclude that transcription is not required in *cis* for origin activation at *DAFC-62D*.

How is it possible that *DAFC-62D* uniquely among the amplicons requires transcription for late stage origin firing, yet this transcription is not required in *cis*? We propose that transcription is required to produce a *trans*-acting factor that is required specifically for *DAFC-62D* origin firing in the developmental time window after stage 10B. Such an origin-specific, *trans*-acting regulatory factor would add to the growing list of mechanisms by which metazoan origins are regulated, emphasizing the idea that individual origins can exhibit unique and distinct mechanisms of regulation. The diversity in the molecular mechanisms that regulate metazoan origin firing is made evident by several *Drosophila* amplicons. These include a local replication enhancer that activates origin firing at *DAFC-66D* (Orr-Weaver *et al.* 1989), ORC-independent origin firing at *DAFC-34B* (Kim and Orr-Weaver 2011), and local repression of ORC binding and origin activity at *DAFC-22B* (Kim *et al.* 2011). Finally, origin-specific *trans*-acting regulators of origin activity could be cell-type specific, allowing an additional level of regulation of origin activation. Thus, identification of this *trans*-acting factor required at *DAFC-62D* and its mechanism of action will prove extremely valuable in our understanding of metazoan DNA replication initiation regulation.

Our results provide perspective not only on the widespread plasticity in mechanisms that control metazoan origin activation but also on the implications of replication initiation errors in genome instability and cancer progression. Misregulation of origin activation can lead to genome instability due to unreplicated or amplified chromosomal regions (Abbas *et al.* 2013), and genomic studies highlight the frequency of copy number changes in cancer cells (Beroukhim *et al.* 2010). Thus, it is crucial not only to understand at the molecular level the range of mechanisms employed to regulate origin activation in metazoan cells but also to understand to what extent these mechanisms present vulnerabilities in genome stability.
